# Direct Physical Contact between Intercalated Cells in the Distal Convoluted Tubule and the Afferent Arteriole in Mouse Kidneys

**DOI:** 10.1371/journal.pone.0070898

**Published:** 2013-09-05

**Authors:** Hao Ren, Ning-Yu Liu, Arne Andreasen, Jesper S. Thomsen, Liu Cao, Erik I. Christensen, Xiao-Yue Zhai

**Affiliations:** 1 Department of Histology and Embryology, China Medical University, Shen Yang, China; 2 Department of Biomedicine – Anatomy, Aarhus University, Aarhus, Denmark; 3 Key Laboratory of Medical Cell Biology, Ministry of Education, China Medical University, Shen Yang, China; 4 Institute of Nephropathology, China Medical University, Shen Yang, China; University of Kentucky, United States of America

## Abstract

Recent physiological studies in the kidney proposed the existence of a secondary feedback mechanism termed ‘crosstalk’ localized after the macula densa. This newly discovered crosstalk contact between the nephron tubule and its own afferent arteriole may potentially revolutionize our understanding of renal vascular resistance and electrolyte regulation. However, the nature of such a crosstalk mechanism is still debated due to a lack of direct and comprehensive morphological evidence. Its exact location along the nephron, its prevalence among the different types of nephrons, and the type of cells involved are yet unknown. To address these issues, computer assisted 3-dimensional nephron tracing was applied in combination with direct immunohistochemistry on plastic sections and electron microscopy. ‘Random’ contacts in the cortex were identified by the tracing and excluded. We investigated a total of 168 nephrons from all cortical regions. The results demonstrated that the crosstalk contact existed, and that it was only present in certain nephrons (90% of the short-looped and 75% of the long-looped nephrons). The crosstalk contacts always occurred at a specific position – the last 10% of the distal convoluted tubule. Importantly, we demonstrated, for the first time, that the cells found in the tubule wall at the contact site were always type nonA-nonB intercalated cells. In conclusion, the present work confirmed the existence of a post macula densa physical crosstalk contact.

## Introduction

One classic mechanism for control of the glomerular filtration rate (GFR) is the tubuloglomerular feedback at the macula densa (MD). Upon increases in tubular NaCl concentration, the afferent arteriole (Af-Art) constricts resulting in a decrease in GFR [1], [2]. Thus, the Af-Art plays an essential role in determining GFR and accounts for most renal vascular resistance [3], [4]. Recently, a post-MD ‘crosstalk’ has been proposed as a secondary feedback mechanism between the Af-Art and the nephron tubule [5]. Ren et al. performed a physiological study with microdissected nephrons from rabbits and demonstrated that an increased sodium concentration of the perfusate in the connecting tubule resulted in dilation of the Af-Art [6]. Morsing et al. performed *in vivo* micropuncture studies in rats, and found that alterations in the flow rate in the distal tubule changed the diameter of the Af-Art [7]. These physiological findings suggest a secondary feedback mechanism localized after the macula densa, which may have great impact on the regulation of glomerular hemodynamics and electrolyte balance in the kidney. However, currently direct morphological evidence for the existence of such a crosstalk mechanism is lacking. In the cortical thick ascending limb [8] and efferent arteriole [9] for instance, additional feedback regulations have also been suggested to exist in some nephrons. Therefore, a comprehensive and systematic survey of such crosstalk is imperative.

The main challenge in the study of such vascular tubular relationships is that it is difficult to distinguish between the thin tubular elements and the small blood vessels. Therefore, it was not until 1992 before Dørup et al. could prove the existence of a direct physical contact between the arterioles and the distal convoluted tubule (DCT) in rat kidneys using computer assisted tubule tracing [10]. Although only a limited number of nephrons were examined, and although the nephrons were only partially traced in the cortical region, the tracing method is still good for reference compatible with the crosstalk study. In the present study, with the aid of modern computer, investigation of large numbers of nephrons becomes possible. Crosstalk contacts can be precisely identified, classified, and localized with the whole nephron tracing. However, so far no such study has been carried out in mice, a species that is convenient for physiological studies and gene manipulation.

The Af-Art originates from interlobular arteries and feeds into the glomerulus [11]. At the juxtaglomerular apparatus, bioactive substances such as adenosine are released from macula densa cells in response to changes in Na^+^ concentration in the tubular fluid causing constriction of the Af-Art [12]. At present, it is not clear whether similar mechanisms are involved in the proposed crosstalk mechanism. Using electron microscopy (EM) and direct immunohistochemistry on plastic sections, we can further identify the cells at the contact site on the sections selected based on the tracing result. This will provide valuable information for further studies of the mechanisms involved in the crosstalk.

## Materials and Methods

### Ethics Statement

All animal experiments were carried out in accordance with provisions for the animal care license provided by the Danish National Animal Experiments Inspectorate. The animals were provided with pathogen-free water and food for maintenance and caged in a controlled environment with a 12/12-h light/dark cycle. The mice were sacrificed by cervical dislocation.

### Preparation of LM serial sections

Kidneys from three adult male C57/BL/6J mice weighing approximately 25 g were studied. In order to avoid deformation during the preparation, the serial sections for 3D (three-dimensional) tracing were obtained from plastic embedded kidneys instead of conventional paraffin wax as previously described in detail [13], [14]. Briefly, the kidneys were fixed by perfusion through the abdominal aorta with 1% glutaraldehyde in 0.06 M sodium cacodylate buffer and 4% hydroxyethyl starch. Tissue blocks were cut perpendicular to the longitudinal axis of the kidney, and post-fixed for 1 hour in 1% OsO_4_ before being embedded in Epon 812. From each kidney, a total of 2,500 2.5-µm-thick consecutive sections were obtained from the surface to the papillary tip and stained with toluidine blue.

### Image recording and aligning

Every second section was digitized using an Olympus AX70 microscope equipped with a×2 objective and a digital camera (Olympus DP 50, Olympus, Tokyo, Japan). The image size was 2,596×1,889 pixels, where each pixel corresponded to 1.16 µm ×1.16 µm. The images were aligned by an established alignment algorithm as previously described in detail [13]–[16]. In brief: the relative transformation values were determined between two consecutive images and then summarized into a set of absolute transformation values. The absolute transformation values underwent a high-pass filtration to avoid potential distortions of the image stacks as a result of small but accumulating “trends”. Then, the images were “moved” according to these absolute transformation values.

### Nephron tracing and length measurements

The tracing of the nephron paths in 3D space was performed on a Linux based PC using custom-made software as previously described in detail [14]. During tracing digital images of the tissue sections were presented on the computer screen and the tubule path was demarked interactively by manually placing markers with the computer mouse on the tubule cross section of the tubule being traced. Tubular tracing was started at the urinary pole of the glomerulus and ended at the collecting duct. The Af-Arts were traced from where they originated from the interlobular arteries until they drained into the glomerulus.

The length of a nephron segment or an Af-Art was calculated as the sum of the Euclidian distance between two subsequent markers in the course of the nephron segment. In order to reduce measurement noise arising from the alignment procedures and the manual placement of the markers, the path was smoothed using a triangular moving average window in such a way that the previous point in the path was weighted with one fourth, the current point with one half, and the next point in the path with one fourth.

### Electron microscopy

Based on the computer assisted 3D tracing, the sections representing the T-A contact sites were selected for ultrastructural analysis. These sections were re-embedded in Epon 812. After polymerization, the resin containing capsule attached to the section was lifted from the glass slide. Then 50 to 70-nm-thick ultra-thin sections were cut before being inspected in an electron microscope (FEI CM-100 electron microscope). The T-A contact sites identified with the computer assisted 3D tracing were first inspected at low (×5,000) and then at high (×15,000) magnification.

### Immunohistochemistry

Immunohistochemistry was performed on the plastic embedded sections as previously described in detail [17]. Briefly, 2.5-µm-thick sections from areas where T-A contacts had been identified were re-embedded in Epon 812. Semi-thin 1-µm-thick sections were cut from these blocks and treated in Maxwell solution (potassium methanol hydroxide) in order to remove the epoxy resin, followed by antigen retrieval in a microwave oven. The sections were then incubated overnight at 4°C with the primary antibody 1∶2000, rabbit a-mouse pendrin (kindly provided by Carsten Wagner, Institute of Physiology, University of Zurich, Switzerland) or 1∶50, sheep anti-mouse renin; 1∶50, rabbit anti-mouse kinin; or 1∶50, rabbit anti-mouse kallikrein (Santa Cruz Biotechnology, Dallas, TX). After washing, the sections were incubated for 1 hour at room temperature with horseradish peroxidase coupled goat anti-rabbit, 1∶250 (Dako, Glostrup, Denmark) or rabbit anti-sheep (Abcam, San Diego, CA, USA). The reaction was visualized with diaminobenzidine. Sections were counterstained with Mayer's haematoxylin.

## Results

In the present study, a total of 127 short-looped nephrons (SLN) and 41 long-looped nephrons (LLN) from the three mouse kidneys were traced and 3D reconstructed, together with their associated afferent arterioles.

### Tubule-afferent arteriole contact

LM inspections reveal that contacts between tubules and arteriole are not rare. However, in the present study, only a contact between the DCT and the Af-Art from the same parent nephron was considered as a positive T-A contact.

**Figure 1:**
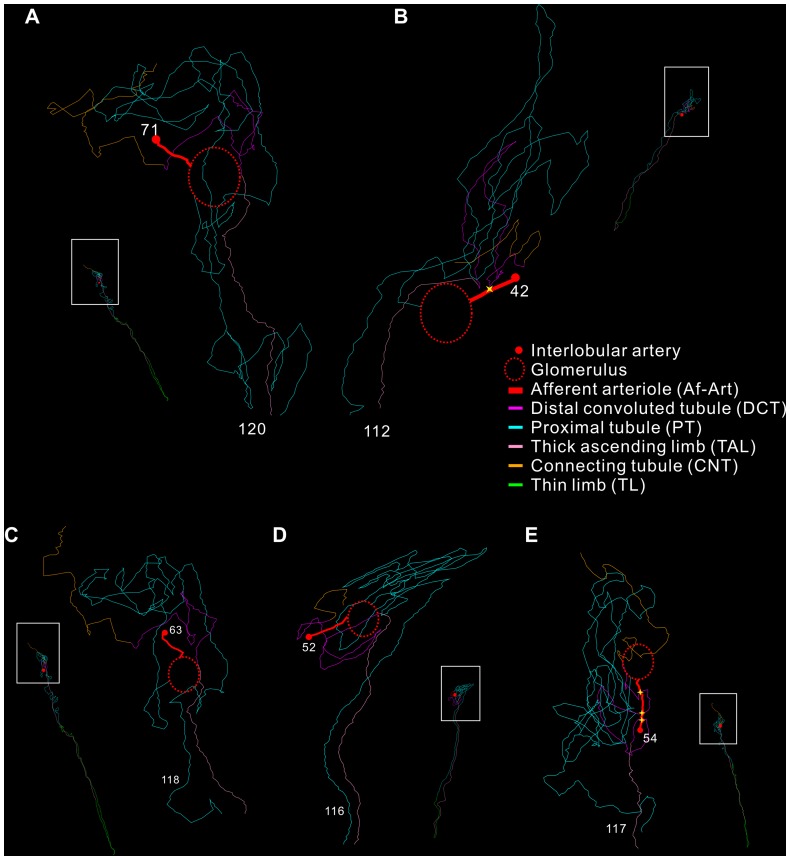
3D plots of various types of physical contacts between the Af-Art and DCT. In each image, there is a whole nephron 3D plot, and an enlargement of the framed area (see notations in (B)). (A) and (B) show single T-A contact between Af-Art (red, tracing Nr. 71 and 42) and DCT (purple, tracing Nr. 120 and 112) of the same nephron. (C), (D), and (E) show cases of no T-A contact, double, and triple (yellow stars) T-A contacts, respectively.

**Figure 2:**
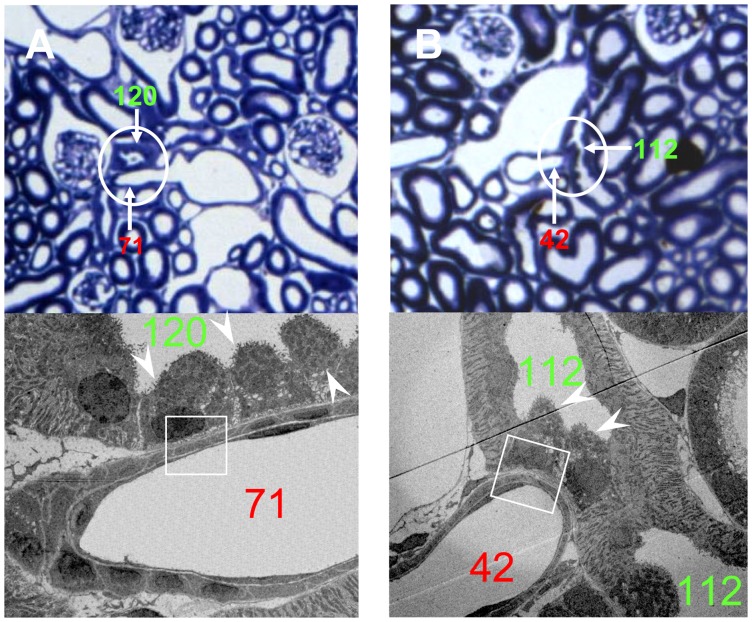
Corresponding LM and EM micrographs of the single contact A and B in Figure 1. (A) show Af-Art (Nr. 71) with tubule (Nr. 120); (B) show Af-Art (Nr. 42) and tubule (Nr. 112). The top images are LM from the corresponding plastic sections stained with toluidine blue. The bottom images are EM from the framed areas in the LM. Note the aggregation of intercalated cells in the DCT at the T-A contact sites (white arrowheads). LM ×400; EM ×5000.

**Figure 3:**
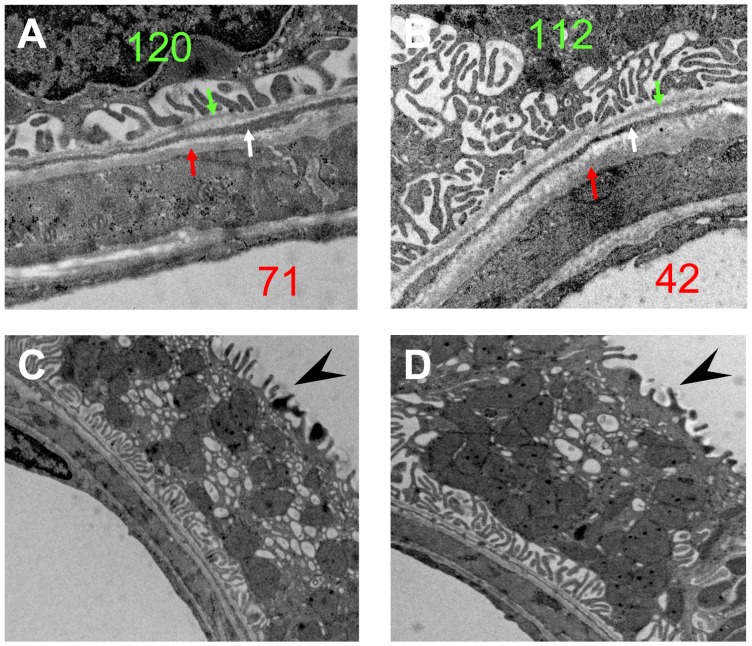
EM observations on the nonA-nonB intercalated cells at contact sites. (A) and (B) are the high magnification images of the framed areas in Figure 2 (nephron Nr. 120 and Nr. 112). The fibroblast extensions (white arrows) interpositioned between the basement membranes of the Af-Arts (red arrows) and the tubules (green arrows). Note the membranous infolding structure on the base of the IC. (C) and (D) are other two examples of the intercalated cells at the contact sites. Note the microprojections on the luminal surface (arrow heads) and the numerous mitochondria and vesicles that are not restricted to the apical area of the cell. (A), (B) ×15,000; (C), (D) ×10,000.

According to this criterion, the tracing data demonstrated that approximately 90% of the SLN had a T-A contact (Table 1). Most of these SLN had only a single contact with the Af-Art (Figure 1A and B). However, in a few cases, double or triple contacts were also detected along the course of the tubule (Figure D and E). In the LM images, the T-A contacts appeared morphologically variable. Frequently, the Af-Art and DCT briefly touched each other and then separated immediately (Figure 2A), but it was also observed that they ran parallel to each other for a short distance. Occasionally, the arteriole crossed or was embraced by the tubule (Figure 2B). EM investigations at the same location from the same re-embedded plastic section revealed that the contacting structures were frequently separated by a long and thin fibroblast extension interposed between the tubular and vascular basement membrane (Figure 3A and B).

**Table 1:** The proportion of T-A contacts in both types of nephrons.

	Traced SLN	Contact(+) SLN	Contact%	Traced LLN	Contact (+) LLN	Contact%	Average contact% per kidney
K1	41	38	92.7	26	21	80.8	88.1
K2	44	40	90.9	6	5	83.3	90.0
K3	42	35	83.3	9	5	55.6	78.4
Sum	**127**	113		**41**	31		
Average			**89.0**			**75.6**	85.7

Approximately 75% of the LLN had a T-A contact (Table 1). The morphology at the contact sites was similar to that of the SLN both at the LM and the EM level. Interestingly, the LLN without T-A contacts were juxtamedullary nephrons, which had a large renal corpuscle located close to the cortical-medullar border. The efferent arteriole of these juxtamedullary glomeruli formed horse-tail descending vasa recta in the medulla.

### Length measurements

The measurement showed that most of the contact sites were located within the last 10% of the DCT for both the SLN and LLN (Figure 1A and B). If there was more than one T-A contact site, the last T-A contact was always located in this last 10% of the DCT (Figure 1D and E).

The T-A contact site on the Af-Art seemed to occur randomly, i.e. the DCT contacted the Af-Art at any place along the course of the Af-Art. Approximately half of the nephrons without T-A contacts had a very short Af-Art (15–40 µm long). Some of these nephrons did not contact their Af-Art. Instead, they contacted either their upstream interlobular artery or the Af-Art of an adjacent glomerulus (from the same nephron family that drained into the same collecting duct). If these “atypical” T-A contacts were also taken into consideration, the percentage of T-A contact positive nephrons were approximately 95% for SLN and 90% for LLN. In addition, most of these “atypical” contacts also occurred within the last 10% of their DCT (data not shown).

### Intercalated cells

**Figure 4:**
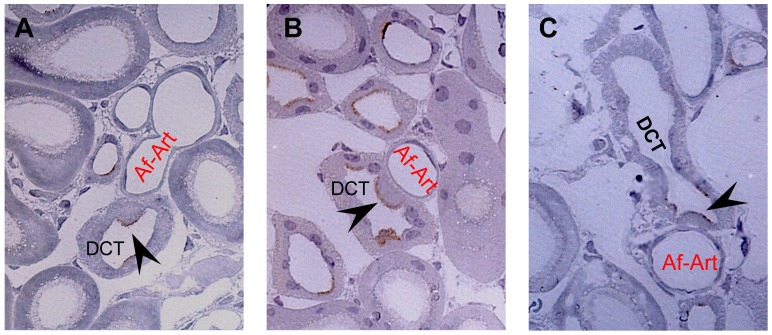
Immunohistochemistry for pendrin in the intercalated cells at the T-A contact site. At the physical T-A contact site of the DCT and Af-Art, the labeling for pendrin was confined to the apical surface of the intercalated cells (arrow heads), which bulged into the lumen of the DCT. The IC were located between DCT cells at the T-A contact site. In each image, the afferent arterioles and the attached DCT belong to the same nephron. ×1000.

Using LM, we always observed a group of cells bulging into the lumen of the tubule at the T-A contact site (Figure 2A and B). This phenomenon was not obvious downstream or upstream on the distal tubule, although such cells were observed individually. We randomly selected six sections containing T-A contact sites for further EM studies. The EM data demonstrated that the bulging cells at the contact sites morphologically resembled intercalated cells (IC, Figure 2 lower panels). These morphological characteristics were similar for all the six sections. The cells appeared identical in size and shape, and often congregated into a group at the contact site. High magnification EM images showed that these IC probably were type nonA-nonB IC. Firstly, the apical surface of the cell was covered by many microplicae, not seen in type A IC. At the same time, the intracellular membrane-bound vesicles distributed throughout the cytoplasm, were not only restricted to the apical area (Figure 3C and D). Furthermore, extensive membranous infolding structures were present at the basal part of the cells (Figure 3A and B). These morphological features are typical of type nonA-nonB IC. Finally, immunohistochemistry on the plastic sections demonstrated the expression of pendrin (Figure 4). In all sections analyzed, the immunolabelling for pendrin was positive on the apical surface of the IC at the T-A contact site, which further confirmed that the cells at the T-A contact sites were type nonA-nonB IC since type A IC do not, or only to a very limited extent, express pendrin. No specific positive staining for either renin, kinin, or kallikrein was observed on the IC at the T-A contact site (data not shown).

## Discussion

In the renal cortex, physical contacts between tubules or tubules and arterioles are frequently observed. Recent functional studies indicated that behind these wall-to-wall contacts, another feedback mechanism may exist in addition to that at the MD [6], [7]. Therefore, it is intriguing to know whether this kind of crosstalk contact is universal or restricted to a specific nephron population. In the present study, we investigated 168 nephrons covering all kidney regions. T-A contacts were observed in almost all SLN (if the “atypical” contacts were also taken into account as described in the Result), and less frequently observed in LLN. The LLN without T-A contacts were juxtamedullar nephrons with large glomeruli giving rise to the horse-tail descending vasa recta that are involved in the establishment of the concentration gradient in the medulla [18], [19]. This result is in line with the fact that the juxtamedullar nephrons are developed during a different phase of the embryonic period than the superficial and mid-cortical nephrons [20], and thus a possible explanation for why the crosstalk mechanism may have developed in one population of nephrons and not in another.

It should be noted that the computer assisted digital tracing of the nephrons and the vessels made it easier to identify “real” T-A contacts as it made it possible to exclude cases were blood vessels and tubular elements were in close proximity of each other without making a direct physical contact.

It is intriguing that the T-A contact always occurred in the last 10% of the DCT. This was also the case for the nephrons that had more than one T-A contact site on the DCT, where the last T-A contact also occurred within these 10%. These data are consistent with the existence of a crosstalk feedback. The MD is located at the terminal of the thick ascending limb, and senses the salt concentration in the distal nephron fluid from the medulla to regulate glomerular filtration [21]. Likewise, it is logical for an additional regulation mechanism to sense the nephron fluid at the end of the DCT, so it can detect changes (in salt concentration or pH) in the tubular fluid that have taken place over the proceeding 90% of the DCT. However, it should be noted that the possibility of similar contacts on other locations (e.g. at the efferent arteriole) is not excluded. Also, the difference of T-A contact between our study and others might also be due to the possible differences among species.

The last 10% of the DCT (also referred to as the DCT2 segment) is where the IC start to appear, and the IC continue in the connecting tubule until the end of the collecting duct [22]. The connecting tubules that contacts the Af-Art as reported by Barajas et al. are perhaps in fact DCT2 segments [23]. In rodents, IC are commonly classified into three subtypes: type A, type B, and type nonA-nonB [24]. Type A IC secrete H^+^ by a vacuolar H^+^-ATPase on the apical cell membrane and express the Cl^−^-HCO_3_^−^ exchanger termed AE1 in the basolateral membrane [25]. Type B intercalated cells secrete HCO_3_^−^ by use of another Cl^−^-HCO_3_^−^ exchanger denoted pendrin, which is located in the apical cell membrane. Pendrin was first identified in patients with Pendred syndrome, a genetic disorder associated with deafness and goiter [25]. Type nonA-nonB IC also express pendrin in the apical cell membrane, but they can be distinguished from type B IC by distinct subcellular structures and distributions. By use of immunohistochemistry for pendrin and EM, we demonstrated that type nonA-nonB IC are always present at the crosstalk contact site. The close association of these IC to the Af-Art was surprising, and strongly suggests that nonA-nonB IC may be part of the crosstalk mechanism. Furthermore, immunohistochemical experiments with renin, kinin, and kallikrein showed no positive signals for these candidates at the contact site, which indicates that the crosstalk regulation may not simply resemble that at the juxtaglomerular apparatus. One possible function of the crosstalk mechanism is that the nonA-nonB IC in the DCT senses certain compositional changes (especially H^+^ concentration) in the afferent arteriole plasma and modulate the downstream type A and type B IC accordingly. There is growing evidence that the various types of IC may represent different functional states of the same cell population, and that these cells change polarity in response to changes in the acid-base status of the animal [26], [27]. Recent studies indicated that pendrin modulates aldosterone-induced changes in ENac abundance by altering luminal HCO_3_^−^ [25], [28]. Changes of the relative abundance of pendrin positive cells were reported with altered distal chloride delivery [29], [30]. Thus, the pendrin positive nonA-nonB IC detected at the contact site may also be a link between acid-base and electrolytes (as NaCl) regulations in the nephron.

In conclusion, the present study suggests that almost all short-looped nephrons and the mid-cortical long-looped nephrons have crosstalk tubular-arteriole contacts in mice. The tubular-arteriole contacts are always present in the last 10% of the distal convoluted tubule, and type nonA-nonB intercalated cells are always present in the distal convoluted tubule at the contact site. These findings may shed new light on our understanding of the feedback mechanism in the nephron, potentially in connection with pH and electrolytes regulation.
